# Evolutionary Roots of Arginase Expression and Regulation

**DOI:** 10.3389/fimmu.2014.00544

**Published:** 2014-11-07

**Authors:** Jolanta Maria Dzik

**Affiliations:** ^1^Department of Biochemistry, Faculty of Agriculture and Biology, Warsaw University of Life Sciences – SGGW, Warszawa, Poland

**Keywords:** arginase, evolution, hemocytes, M1/M2 macrophages, nitric oxide synthase, parasites, TGF-β, wound healing

## Abstract

Two main types of macrophage functions are known: classical (M1), producing nitric oxide, NO, and M2, in which arginase activity is primarily expressed. Ornithine, the product of arginase, is a substrate for synthesis of polyamines and collagen, important for growth and ontogeny of animals. M2 macrophages, expressing high level of mitochondrial arginase, have been implicated in promoting cell division and deposition of collagen during ontogeny and wound repair. Arginase expression is the default mode of tissue macrophages, but can also be amplified by signals, such as IL-4/13 or transforming growth factor-β (TGF-β) that accelerates wound healing and tissue repair. In worms, the induction of collagen gene is coupled with induction of immune response genes, both depending on the same TGF-β-like pathway. This suggests that the main function of M2 “heal” type macrophages is originally connected with the TGF-β superfamily of proteins, which are involved in regulation of tissue and organ differentiation in embryogenesis. Excretory–secretory products of metazoan parasites are able to induce M2-type of macrophage responses promoting wound healing without participation of Th2 cytokines IL-4/IL-13. The expression of arginase in lower animals can be induced by the presence of parasite antigens and TGF-β signals leading to collagen synthesis. This also means that the main proteins, which, in primitive metazoans, are involved in regulation of tissue and organ differentiation in embryogenesis are produced by innate immunity. The signaling function of NO is known already from the sponge stage of animal evolution. The cytotoxic role of NO molecule appeared later, as documented in immunity of marine mollusks and some insects. This implies that the M2-wound healing promoting function predates the defensive role of NO, a characteristic of M1 macrophages. Understanding when and how the M1 and M2 activities came to be in animals is useful for understanding how macrophage immunity, and immune responses operate.

Vertebrate macrophages play an innate defense role against various pathogens. They perform phagocytosis, bacterial killing, defend against protozoan and metazoan parasites and take part in wound healing. To fulfill such protective functions, “resting” macrophages must be activated. Two main types of macrophage functions have been identified: classical (M1), producing nitric oxide (NO), and M2-type, in which arginase (producing the healing molecule, ornithine) is expressed (1, 2). These responses from macrophages demand different cascades of biochemical reactions, which are regulated by specific sets of cytokines. M1 type can be stimulated by pathogen associated molecular patterns (PAMP) or amplified by T cell cytokines, such as IFN-γ. In contrast, M2 activity is the resident tissue type, and can be amplified by molecules such as IL-4, IL-13, and transforming growth factor-β (TGF-β). Local signals polarize macrophages to an appropriate response. These immune functions are indispensible for both life of an individual and lasting of a species. It is apparent that macrophages, as well as other cells of innate immune response acting in vertebrates, have their deep evolutionary roots in cells serving similar function in ancestral invertebrates. Various names have been used to define such cells in different invertebrate groups, i.e., hemocyte, coelomocyte, amebocyte, or plasmatocyte, collectively named immunocytes (3). However, regardless of the terminology, they perform the same immune functions, and are of similar morphology.

Macrophages, the professional phagocytic cells in humans, derive from circulating monocytes or by self renewal in the tissues, and acquire new morphological and physiological characteristics according to the organs and microenvironments, in which they settle. However, this unitarian origin is uncertain for circulating and tissue phagocytes (immunocytes) in invertebrates (3). Immunocytes and macrophages share ability to be activated by non-self material and to react through the release a variety of biologically active molecules such as cytokines, NO, reactive oxygen species, hydrolytic enzymes, and neuroendocrine mediators. In vertebrate immunity, various organs and immune cells are involved, while all the molecules determining invertebrate immune response are harbored in the immunocytes. From the perspective of this review, the multifunctional role of invertebrate cells seems instructive in respect to its inheritance by vertebrates.

In search for selection pressure for macrophage differentiation into M1 and M2 phenotypes, it is tempting to look back to recognize which of the functions of M2 macrophages is aligned straight with invertebrate immunocytes and is found possibly in the most primitive invertebrates. Accumulated evidences strongly suggest that a primary function of M2 macrophages is tissue repair and wound healing (4, 5). This process demands polyamines and collagen synthesis what strongly depend on arginase activity (6).

## Animal Arginases

Arginase (amidinohydrolase, EC 3.5.3.1) is an ubiquitous enzyme found in bacteria, yeasts, plants, invertebrates, and vertebrates. Some bacteria possess a related enzyme, agmatinase (*Escherichia coli* or *Methanobacterium*, also *Methanococcus*), which belong to Archaebacteria. Agmatinase produce putrescine and urea, arginase ornithine and urea. Plant arginases are closer to the agmatinase clade than to the animal one (7, 8). Agmatinases origin predates that of arginases and the latter would have appeared in the Bacteria by recruitment of a wide specificity agmatinase and then its transfer to an eukaryotic cell (according to Sekowska et al. perhaps through mitochondria) (8).

Most microorganisms and invertebrates studied to date have only one type of arginase (9). Arginases from plants and ammoniotelic animals are localized in mitochondria (10). In ureotelic animals, arginase is involved in ammonia detoxication in the ornithine-urea cycle and is localized in cytosol. The cytosolic and mitochondrial arginases are isoenzymes named A-I and A-II, respectively (11). They are encoded by two separate genes. The arginase gene duplication is relatively recent, and occurred after separation of vertebrates and invertebrates (9). It has been suggested that the mitochondrial A-II is a surviving form of the ancestral arginase, because the cytosolic A-I is restricted to a subset of more recently evolved species (11).

The pattern of occurrence of the arginase isoenzymes implies that the primordial function of the enzyme is regulation of cellular arginine and ornithine metabolism, unrelated to the urea cycle. Ornithine, the product of arginase-catalyzed reaction, is a substrate for synthesis of proline and polyamines. It is important to know that ornithine is formed from glutamate *via* the pathway leading to arginine synthesis as well. This pathway occurs in bacteria, plants and animals (10). Both in plants and animals, polyamines (putrescine, spermidine, and spermine) are involved in a variety of growth and developmental processes, and they bind directly to DNA and RNA (12). It has been also shown that polyamines play a pivotal role in wound healing. Proline (as hydroxyproline) is indispensable for the collagen biosynthesis in animals and for the synthesis of cell wall proteins in plants. Participation of ornithine in such vital processes suggests that arginase could be regulated by factors influencing development and growth of organisms. TGF-β belongs to a superfamily of ancient proteins, known in all bilaterians, members of which play important signaling roles in embryogenesis (13).

## TGF-β Superfamily of Proteins

Transforming growth factor-β was originally discovered as a secreted factor that induced malignant transformation *in vitro*. It is a prototype member of a superfamily of secreted, homodimeric polypeptides. These factors affect a variety of biological processes in both transformed and normal cells, including regulation of embryogenesis, adult cell differentiation, inflammation, and wound repair (14, 15).

The TGF-β superfamily may be divided into subfamilies according to sequence homology. One subfamily consists of the closely related TGF-β1, -2, and -3. TGF-β2 and -β3 take part in development signaling, while TGF-β1 signals act in inflammatory responses and tissue necrosis (15). TGF-β1 cDNAs from different animal species (together with chicken and *Xenopus*) show an extremely high degree of conservation (14). Fish TGF-β homologs cluster with their mammalian TGF-β1, -β2, and -β3 counterparts (15).

Bone morphogenetic proteins were initially characterized as factors that induce bone and cartilage formation (16, 17). Bone morphogenetic proteins (BMPs) are critical in development, hematopoiesis, as well as cellular chemotaxis, and cellular differentiation (18, 19). In the BMPs subfamily, BMPs-2 and -4 bear closest homology to the decapentaplegic complex protein, a *Drosophila* protein mediating dorsal/ventral axis specification (14). BMPs-5, -6, -7, and -8 most closely resemble *Drosophila* protein 60A, which is required for the growth of imaginal tissues and for patterning of the adult wing (20). Genes encoding members of the bone morphogenetic factor (BMP) protein family have been identified in a sea anemone and an echinoderm (21).

Genes of TGF-β superfamily members cluster in two major clades: TGF-β sensu stricto/TGF-β related (e.g., Activins, Leftys, and GDF8s) ligands and BMP related (e.g., BMPs and Nodals) (22). TGF-β sensu stricto ligands have been identified only in deuterostomes (Echinodermata, Hemichordata, and Chordata) and are not present in genomic screens of *Caenorhabditis*, *Drosophila*, or *Nematostella* (23).

Receptors of TGF-β pathway are serine threonine kinases categorized as type I and type II (24, 25). Vertebrates have seven distinct type I receptors, each of which can mix and match with one of five type II receptors to mediate signals for the TGF-β family ligands (26). Ligand binding to the constitutively phosphorylated type II receptors stimulates recruitment of type I receptors and formation of a heterodimeric receptor complex. In the complex, type I receptors are transphosphorylated by type II receptors (13). A signal from type I receptor to the nucleus is channeled into one of two intracellular pathways *via* Smad family of proteins. Three of the receptors phosphorylate the R-Smads (receptor-regulated Smads); Smad2 and Smad3 and thereby transduce TGF-β-like signals, whereas the other four receptors activate the R-Smads; Smad1, Smad5, and Smad8 to mediate signals characteristic of those initiated by BMPs (26–28). These R-Smads form multisubunit complexes with a common partner Smads (Co-Smads; Smad4) before entering the nucleus to affect a response (29). Both R-Smads and Co-Smads are found in all metazoans (30). I-Smads; Smad6/7 play inhibitory function, stimulating receptor degradation, or competing with R-Smads in formation of complexes with Smad4 (29, 31). The regulatory activity of I-Smads evolved after divergence of the poriferan lineage (32).

Binding of Smads to DNA is not especially specific; they play a role of comodulators, which act together with transcription factors (pan-metazoan Fos/Jun and Myc), transcription coactivators (pan-metazoan CBP and CBF-β), and transcription corepressors (Ski/Sno) to recruit basal transcription machinery (32). The formation of Smad complexes gives a wide range of cooperative interactions, thus enables TGF-β signaling to evoke multiple responses ranging from embryonic development to wound repair. AR-Smads (activin/TGF-β-specific R-Smads) transactivate various target genes through interaction with various DNA-binding partners, including plasminogen activator inhibitor-1 (PAI-1), type I collagen, junB, Smad7, and Mix.2. For inhibition of cell growth by TGF-β, AR-Smads induce the transcription of cyclin-dependent kinase (CDK) inhibitors p21 and p15. In addition, Smad3 binds directly to the promoter region of c-myc and represses the transcription of c-myc. In contrast, only a few target genes for BMPs have been identified, including Id (inhibitor of differentiation or inhibitor of DNA-binding) 1/2/3, Smad6, Vent-2, and Tlx-2. Id proteins, however, play important roles in multiple biological activities of BMPs (27). Id proteins act as negative regulators of cell differentiation and positive regulators of cell proliferation (33).

Ligands, receptors of the TGF-β pathway, and Smads are ancient proteins. They emerged already in the metazoan stem lineage. I-Smads, multiple ligand traps, and SARA have been added to the signaling pathway after the divergence of sponges (32).

Transforming growth factor-β and BMP signaling pathway is evolutionary conserved, as it was shown for worms, flies, and vertebrates [Ref. (26)].

## TGF-β Function in Embryogenesis and Wound Healing of Invertebrates

*Caenorhabditis elegans* is a free-living nematode, a member of the lineage, which appeared more than 470 million years ago (34). There are at least three distinct TGF-β-like pathways in this worm (35). One of them controls the body size and morphology of the male tail, but five genes of this pathway (*dbl-1, sma-2/-3/-4/-6*) contribute to resistance against *Pseudomonas aeruginosa* infection (36). This pathway controls induction of some genes induced after *Serratia marcescens* infection, including *lys-8* (lysozyme) (37). Moreover, *dpp*, a *dbl-1* homolog in *Drosophila*, is up-regulated upon immune challenge (38, 39). This pathway shows a clear homology to the mammalian TGF-β pathway (40), which plays an important role in immune responses (41). Some targets of this pathway in *Caenorhabditis* are known: *mab-21*, involved in male ray pattern formation, and *lon-1* and *lon-3*, involved in regulation of the body size. The latter two encode a cysteine-rich secretory protein and collagen, respectively. The *lon-1* and *lon-3* are mainly expressed in the hypodermis, as they are essential for the body size regulation (42, 43).

This finding implies that the ancestral pathway of TGF-β signaling in embryogenesis is bound with immune reactions in the Protostomia, suggesting that TGF-β pathway in immunity has been conserved generally across their evolution. Induction of collagen gene in *Caenorhabditis* during bacterial infection links developmental processes with the tissue repair induced by pathogens. Collagen is needed for extracellular matrix deposition during both embryogenesis and wound repair. Hemocytes must migrate to wounded area for the synthesis of collagen fibrilles. In lesioned leeches (Annelida), immunocytes are the first cells that are also involved in closing the wound by using pseudopodia to bridge the epithelial edges. Subsequently, additional immunocytes complete the obstruction together with granulocytes and NK-like cells (3). Throughout embryogenesis, hemocytes carry out important developmental functions within the embryo, such as the engulfment and removal apoptic cells and the laying down of many extracellular matrix molecules, including collagen IV and laminin, which compose the basement membrane surrounding internal organs [Ref. (44)]. *Drosophila* hemocytes are similar to leukocytes in respect of activation and migration toward wounds. A requirement for phosphoinositide 3-kinase (PI3K) for the polarization and active hemotaxis of hemocytes toward an epithelial wound shows a striking analogy with the mechanism of cell chemotaxis used by *Dictyostelium discoideum*, mammalian neutrophils (45), as well as fish macrophages (46). Migration of *Drosophila* hemocytes toward wounds depends on CDC42 and Rho signal transmission (47) and PI3K signaling (45). The migratory pattern of hemocytes in *Drosophila* embryos is independent of PI3K signaling, but depends on chemotactic signals from the PDGF/VEGF ligands (45).

During wound healing in the snails *Lymnaea stagnalis* (48) and *Limax maximus* (49), hemocytes exhibit fibroblast activity while secreting the extracellular matrix. Similar transformation of a cell type was observed in fibroblasts transformed into myofibroblasts at injured sites when acting in wound contraction (50). It points to the role of hemocytes in invertebrate tissue repair, similarly to the mammalian wound models, in which the macrophages are essential cells participating in wound healing (51). PDGF-AB (hetrodimer) and TGF-β1 stimulate chemotaxis of different cell types, especially hemocytes (49). The increased number of hemocytes contribute to earlier wound closure at the injured site. The removal of damaged tissue residues is also accelerated by stimulation of the phagocytic activity of recruited hemocytes. TGF-β1 regulates expression of genes of collagen type I and III, and fibronectin (52, 53). This may mean that the mechanism of wound healing is conserved from invertebrates to mammals what’s more, arginase gene expression has been documented in hemocytes (54).

Tissue injury in humans triggers migration of macrophages, platelets, fibroblasts, myofibroblasts, and eosinophils releasing TGF-β. TGF-β stimulates fibroblasts and other reparative cells to proliferate and synthesize extracellular matrix components (elastin fibers, collagen fibrils, protein–polysaccharides, and glycoproteins). This leads to a provisional repair, followed by fibrosis and ultimately scarring. Fibrosis of many organs (liver, heart, kidney, pancreas, and skin) is mediated by TGF-β [Ref. (55)]. In experimental murine *Schistosoma mansoni* infection, gene expression of type I and type III interstitial collagens, basement membrane collagen, and TGF-β1 show increased levels of expression after primary infection (56). Transcription of type I procollagen chains *pro*α*1* and *pro*α*2* is TGF-β-regulated through two different pathways during tissue fibrosis. Expression of *pro*α*1* depends on the TGF-β activator protein and expression of proα2 depends on Smad signaling of TGF-β pathway. In addition, there are other cellular factors and DNA-binding elements required for the transcription of these type I procollagen genes. New synthesized procollagen molecules are processed by enzymes outside the cell.

In the evolution of nematodes, they changed their original free-living habitus to commensal one and finally to parasitize tissues of animals (57). The evolution of plathelminths was different. They changed ectoparasitic mode of life of monogenean trematodes to endoparasitic one of digeneans and tapeworms. Invertebrates that are parasitized by these worms, heal damages to the tissue due to activation of polyamine and collagen synthesis. Thus arginase induction required for wound healing in animals without acquired immunity could be based on the TGF-β signaling pathway.

Aside being a substrate for proline synthesis pathway, ornithine is a substrate for polyamine production in all eukaryotic cells. As polyamines are required for high rates of protein synthesis and cell proliferation (58), they play a pivotal role in repair processes. Stimulation of putrescine synthesis was observed during regeneration of earthworms and planarians (59). Regenerating tissues produce spermine, and injured or dying cells release spermine into the extracellular milieu, so that tissue levels of this compound increase significantly at inflammatory sites of infection or injury (60). In snails resistant to the *Schistosoma mansoni* infection (61), increased gene expression of ornithine decarboxylase in hemocytes points to the enhancement of arginase activity, which results in ornithine production (62, 63). Ornithine decarboxylase produces putrescine used for the synthesis of other polyamines involved in DNA protection during cell proliferation. Polyamines assist in wound healing following miracidial penetration.

Mollusks can be infected with viruses, bacteria, fungi, protists, digenean trematodes, polychetes, and copepods (64). The infective stage of the protozoan (haplosporidian) *Haplosporidium nelsoni* invades the bivalve tissues through gills and palps spreading then through the body. Infection of the bivalve *Crassostrea virginica* with this protist leads to an increase in the number of circulating hemocytes and their infiltration into tissues (65). It is suggested that these cells are involved in limiting parasite damage by plugging lesions, removing debris, and repairing damaged tissue.

In hemocytes of the snail *Biophalaria glabrata* infected with the digenean trematode *Schistosoma mansoni*, for which a definitive host is human, the expression of TGF-β receptor gene was slightly lowered in comparison with those of resistant strains. Early gene expression was measured only 2 h post exposure to miracidia (61).

Transforming growth factor-β signaling is essential for extracellular matrix development in cold-blooded animals (66). As a result of infection of salmonid fish with the ectoparasitic caligid crustacean *Lepeophtheirus salmonis* insufficient expression of several regulatory proteins, among them TGF-β, brought up delayed expression of collagen 2a and delayed wound healing. Arginase gene expression was markedly increased in intact skin of infected fish (67). Support for the hypothesis that arginase expression is related to collagen expression comes from observations that arginase trancripts are down-regulated in concert with collagen a in resistant oysters five days after challenge with the gram-negative bacterium *Roseovarius crassostreae* (68). This extracellular pathogen colonizes the oyster’s inner shell surface and causes lesions in the epithelial mantle.

Efficient wound healing in invertebrates based on induction of genes for arginase and collagen biosynthesis (68) mediated by TGFβ (49, 65), but without cytokines of Th2 cluster being involved, may mean that also in vertebrates such mechanism of healing is possible. An innate response to injury may occur in absence of any adaptive response and can be triggered solely by tissue injury (69). Although IL-4/IL-13 mediated responses may be important in tissue repair, they do not appear to be essential, as the incision is effectively healed in the mice that lack IL-4 or IL-4 receptor. Nonetheless, the importance of type 2 cytokines in damage tissue remodeling and fibrosis is well documented (70). Possibly IL-4 and/or IL-13 mediate a more rapid form of tissue repair that it is necessary just to maintain tissue integrity. According to Allen and Wynn (71), Th2 immunity in vertebrates evolved as a means to rapid tissue damage repair caused by metazoan invaders rather than just to control parasite numbers.

## M2-Type of Macrophage Response without Help of Th2 Cytokines

Transforming growth factor-β, IL-4, and IL-13 are key cytokines skewing macrophages to the M2-type response that is typical for allergy and metazoan parasite infection. Arginase induction is the hallmark of this response. This raises the question whether M2-type of macrophage response could be induced solely by multicellular parasites without help of Th2 cytokines.

A strong wound healing response would occur in helminth infection, as tissue migratory or tissue invasive parasites often lead to physical trauma. A Th2-type protective immune response develops following infection with many tissue-dwelling intestinal nematode parasites (*Heligmosomoides polygyrus*, *Trichuris muris*, or *Trichinella spiralis*) or trematodes and is characterized by elevations in IL-4 and IL-13 and increased numbers of CD4^+^ T cells, granulocytes, and macrophages. These cells accumulate at the site of infection and may mediate resistance to worms (72, 73). This is a kind of defense strategy of the host, but it eventually favors a survival of parasite.

Excretory-secretory (ES) products or parasite enzymes activate and regulate host-immune response at the macrophage level through inhibition of pro-inflammatory cytokines production and induction of macrophages toward the M2-type of activation.

*Trichinella spiralis* is the parasitic nematode of higher vertebrates, which causes pathological changes in various tissues of the host. Binding of TGF-β with specific antibodies abrogated effect of infection on arginase activity in guinea pig alveolar macrophages (74). ES products from *Trichinella spiralis* raise the expression of interleukin-10, TGF-β, and arginase-1 in J774 A.1 macrophages in the absence of Th2 cytokines (75). In addition, ES products significantly inhibit translocation of NF-κB into the nucleus and the phosphorylation of both ERK1/2- and p38MAP-kinases in J774A.1 macrophages stimulated with lipopolysaccharide (LPS) (an antigen of Gram-negative bacteria). Treatment of peritoneal macrophages with a recombinant of 53-kDa protein derived from *T. spiralis* brought about expression of mannose receptor, a novel mammalian lectin (Ym1), arginase-1, and IL-10, hallmarks of M2 phenotype. This effect was independent of IL-4Rα, but dependent on STAT6 (76).

Infections with the trematodes *Fasciola hepatica* or *Schistosoma mansoni* cause destruction of the host liver tissues, damage to bile ducts, atrophy of the portal vessels, and secondary pathological conditions. Secreted peroxiredoxins may induce alternative activation of macrophages. They stimulate Ym1 expression *in vitro*, which shows their action independent of IL-4/IL-13 signaling (77). As expected, administration of recombinat peroxiredoxins from these trematodes to the wild type and IL-4^−^/^−^ and IL-13^−^/^−^ mice induces alternatively activated macrophages. Also eggs of *S. mansoni* laid in the smallest blood vessels cause tissue reaction in the form of inflammation, necrosis, connective tissue encapsulation, and eventual scar formation during their migration through the tissue to the colon. The eggs trapped in the liver induce fibrosis and are associated with production of proline (78). The immunomodulatory pentasaccharide LNFPIII, which contains the Lewis X trisaccharide, is a component of schistosome soluble egg antigen. It up-regulates expression and activity of arginase-1, as well as expression of Ym1 in macrophages (79) but does not induce expression of FIZZ-1, MGL-1, or MMR. Upregulation of arginase I and Ym1 is independent of IL-4 and IL-13. Binding of LNFPIII to C-type lectins on the surface of macrophages leads to alternative nuclear factor (NF)-κB activation (80) and may induce arginase-1 and Ym1 directly without IL-4 and IL-13. An injection of LNFPIII initiates alternative activation, do not mimicking complete infection because it does not cause FIZZ-1 expression, besides upregulation of Ym1. Interestingly, Loke et al. (69) have found that surgical trauma leads to elevation of markers of alternative activation without presence of T cells. However, the innate expression of Ym1, FIZZ-1, and arginase-1 requires either IL-4 or IL-13. Expression of arginase-1 occurred early in response to surgery. It increases with growing up to third day post surgery and then returns to baseline by 1 week, but is sustained only in the parasite-implanted animals.

Protozoan parasite *Toxoplasma* type I and type III strains may induce the M2 phenotype, while the type II strain induces M1 phenotype (81). The alternative activation of macrophages is dependent in large part on the *Toxoplasma* polymorphic protein kinase ROP16, while the classical activation of macrophages by the type II strain is due to unique ability of its GRA15 protein to activate NF-κB pathway and elicit pro-inflammatory cytokines. Both enzymes seem act in a way specific to the host. According to authors, parasite effectors from different *Toxoplasma* strains evolved to work optimally in hosts predisposed to certain types of immune responses, such as those along the Th1/Th2/Th17 or M1/M2 axes. Ending up to the wrong host might lead to severe disease and failure to establish chronic infection.

## Macrophage Polarization by TGF-β Superfamily Proteins

In the adult *Drosophila* immune response, *Dpp* (*decapentaplegic)*, a BMP-type signal, is rapidly activated by wounds and represses the production of antimicrobial peptides. The activin/TGF-β-like signal *dawdle* (*daw*), in contrast, is activated by Gram-positive bacterial infection but repressed by Gram-negative infection or wounding; its role is to limit infection-induced melanization. Genes *dpp* and *daw* are expressed in hemocytes but also in other tissues. The hemocyte population in the adult fly is comprised of subsets of cell that can be defined through distinct gene expression profile. According to Clark et al. (82), it is likely that expression of *dpp* and *daw* by a subset or subsets of phagocytes indicates distinct immunomodulatory functions by these cells. Both *dpp* and *daw* inhibit immune responses. This makes the fly similar to mammals, in which both activin and TGF-β-like and BMP-like signals are largely anti-inflammatory (83, 84), in contrast with the nematode *Caenorhabditis*, where the TGF-β superfamily member *dbl-1* analog of *dpp* promotes a variety of antimicrobial responses (85).

Both anti- and pro-inflammatory response due to activation of TGF-β superfamily receptors by their ligands, TGF-β, bone morphogenetic protein-7 (BMP-7), BMP-6 have been found in macrophages of rodents. Surprisingly few studies have evaluated the effect of TGF-β signaling on macrophages. Mouse macrophages lacking TβRII (transforming growth factor-β receptor II) are defective in expression of genes that characterize the M2-type of activation, suggesting that TGF-β signaling is needed for the alternative activation of macrophages. Lack of *T*β*RII*^−/−^ is associated with basal expression of arginase-1 (protein and mRNA) significantly decreased in comparison with the wild type both in naïve peritoneal macrophages and bone marrow-derived macrophages, BMDM, (86). Moreover, when *T*β*RII*^−/−^ BMD macrophages are polarized toward an M2 phenotype with IL-4, induction of Arg-1 is very low. Expression of Arg-1 is increased in *WT* macrophages stimulated with TGF-β1. As transcription of other M2 markers including *ym1* ceases in *T*β*RII*^−/−^ BMDMs, apparently signals through TβRII modulate the M2 transcription program. TGF-β contributes to M2 polarization of macrophages with IL-4 through co-signaling to Akt, which is one of the TGF-β1 non-Smad-associated signal transduction pathways in other cell types (87).

Bone morphogenetic protein-7 activates receptor BMPR2 in monocytes, which results in phosphorylation of R-SMAD1/5/8/and activation of down-stream mediators in the Smad pathway. It plays a role in polarization also in M2 macrophages, as manifested by increased expression of anti-inflammatory cytokines (88). In bone marrow-derived M2 macrophages, increased polarization results from activation of PI3K pathway (89). Activation of the PI3K pathway controls production of transcription factors. They regulate key inflammatory cytokines resulting in increased expression of anti-inflammatory markers (90). Arginase-1 and IL-10 level is significantly increased following treatment of monocytes with BMP-7 (88). In addition to the canonical Smad-dependent pathway for TGF-β signaling, a Smad-independent pathway, namely the mitogen-activated protein kinase (MAPK) pathway (p38MAPK and JNK) may act (91). Activation of the NF-κB pathway via the X-linked inhibitor of apoptosis (XIAP) transduces BMP signaling (92). Different to BMP-7, BMP-6 may induce pro-inflammatory inducible NOS (iNOS) and TNF-α in peritoneal macrophages (93). The general phenotype of macrophages in response to BMP-6 is similar to that of macrophages exposed to LPS (94). BMP-6 in macrophages appears to counteract TGF-β. It is likely that the BMP-6 induction of expression of inducible NO synthase occurs through IL-1β *via* Smad and NF-kappaB signaling pathways (95). IL-1β, in turn, up-regulates iNOS expression *via* the NF-κB pathway. However, the possibility that BMP-6 may directly activate NF-κB signaling could not be excluded because TGF-β activates kinase 1 (TAK1), which is a component of the BMP signaling pathway in *Xenopus* and mouse embryonic development (96, 97). TAK1 and its regulators (TAB1 and TAB2) form complexes and activate the IKK complex (98). The latter possibility suggest that pathway used in embryonic signaling could be used in innate immunity response to induce NO production. Interestingly, NO and ornithine (the product of arginase described earlier), both originate from the same amino acid, arginine, *via* different enzymatic reactions, which were called figuratively “The arginine fork in the road” (99).

## Evolution of Nitric Oxide Synthase

In macrophages, NO is a crucial mediator of cytotoxicity. It has been shown to have microbicidal, antiviral, antiparasitic, and antitumor effects. NO production is usually mediated by nitric oxide synthase (NOS) (EC 1.14.13.39). To date, three isoforms of NOS have been characterized: iNOS, neuronal NOS (nNOS), and endothelial NOS (eNOS). In macrophages, iNOS is transcriptionally induced in response to LPS, TNF-α, interferon-γ (IFN-γ), and interleukin-1β (IL-1β) (100). The signaling pathway for iNOS expression in macrophages involves NF-κB and signal transducer and activator of transcription (STAT).

Three NOS isoforms originally described in mammalian tissues (100, 101) are encoded by distinct genes: for eNOS, nNOS, and iNOS. All they share much of their sequence with cytochrome P450 reductase in their C-terminal reductase domains and have a common oxygenase domain.

The interdomain linker between the oxygenase and reductase domains contains a calmodulin-binding sequence. In eNOS and nNOS, physiological concentrations of Ca^+2^ in cells regulate the binding of calmodulin to the linker, thereby initiating electron transfer from the flavins to the heme moieties. In contrast, calmodulin remains tightly bound to the iNOS (Ca^+2^ -insensitive isoform). Expression of iNOS is strongly activated in the presence of LPSs or in response to potentially damaging stimuli, resulting in a high and long-term NO yield. iNOS is primarily involved in defense reactions and cytotoxicity.

Nitric oxide synthase ancestry goes back to early bacteria from before a couple billion years ago. In all prokaryotic enzymes only the oxygenase domain is found (102, 103). In NOS evolution, multiple events of gene loss and gain in various lineages occurred.

Nitric oxide synthase occurs probably in almost all invertebrates ranging from jellyfish (104) and hydra (105) to fly (106) and parasitic worms (107), as well as mollusks and arthropods (108). NOS enzymes from insects (109–111) and mollusks [(112), Ref. (113)] have greater overall sequence similarity to a neuronal-like NOS than to iNOS or eNOS in vertebrates. However, cnidarian (*Discosoma*) and slime mold (*Physarum)* NOSs (113, 114) lack the distinct structural element that is present as an insertion in the reductase domains of constitutive NOSs but is absent in iNOSs of vertebrates. This insert is thought to be an autoinhibitory loop, which impedes binding of Ca^2+^ to calmodulin and enzymatic activation. This insert reduces potentially toxic NO yields following the activation of iNOS. Since the *Discosoma* NOS is structurally similar both to the only known non-animal conventional NOS and to vertebrate iNOS isoforms, the inducible type of the enzyme may be ancestral for animal NOSs. In contrast to vertebrate species, which have three NOS genes, only one type of NOS isoform has been found in the genomes from insects and tunicates. Mollusks, sea urchins, and cephalochordates have at least two NOS genes but no NOS genes have been identified in *Caenorhabditis elegans*. These findings imply that more than one NOS co-existed in the common ancestor of all animals and it was lost in some animal lineages in the course of evolution. On the other hand, in some groups, such as mollusks (with at least two different types of NOS) and chordates (2–3 NOS genes), duplication events for NOS genes may have occurred more than once. Moreover, duplications happened independently in the evolution of inducible type NOS, since some fishes have more than one iNOS-like gene (113). The diversification of vertebrate NOSs occurred in parallel in many lineages, which cluster into three distinct groups corresponding to the mammalian iNOS, eNOS, and nNOS, iNOS probably being most basal. eNOS apparently originated as the last, within the mammalian clade.

The primary and evolutionary conservative role of NO is NO-cGMP signaling, acting in many different invertebrates from sponges, insects, and mollusks to cephalopods [Ref. (115)]. A defense function of NO was observed in the crustacean (116), and mollusk hemocytes [Ref. (117)]. In general, defense functions in invertebrates are accomplished by superoxide produced by phagocyte NADPH oxidase (which appeared before the divergence of the Choanoflagellata and metazoans), antimicrobial peptides, lysozymes, hemolymph clotting, and melanization [Ref. (115)]. This suggests that the function of arginase as the key enzyme producing ornithine in metazoans, indispensible for tissue repair, is more ancient than the cytotoxic activity of free radical NO, the product of NOS. One may conclude that the wound healing function of M2 macrophages is more deeply rooted in history of life than the cytotoxic activity of M1 macrophages.

## Regulation of Nitric Oxide Production in Insects and Mice

Most of research on the NOS refers to three animal species: fruit flies *Drosophila melanogaster*, mice *Mus musculus*, and humans *Homo sapiens*. Presumably, the insects are the least advanced its evolution. Mice, as rodents, are relatively primitive mammals, closest relatives of the order Primates, to which humans belong. From evolutionary point of view, the rodent macrophages, commonly used as a model for immunological investigations, better suit to studies on innate immunity reactions in vertebrates than human ones.

The model for studying arthropod immunity is the antimicrobial defense in *Drosophila*. IMD pathway performs a signaling function by inducing host defenses in response to Gram-negative bacteria. Activation the IMD signaling pathway leads to the activation of NF-κB homolog Relish and production of antimicrobial peptides (118, 119). The cells in *Drosophila* gut detect the pathogen and activate hemocytes *via* an NO-dependent signal. The hemocytes act in turn to activate immune-inducible gene expression in the fat body (the insect liver analog) by an as-yet-unknown signal (118). In *Anopheles stephensi*, expression of immune responsive genes, including NOS, is up-regulated in response to the presence of *Plasmodium* parasites in the midgut (120).

It has been found that *A. stephensi* NOS possesses a putative LPS- and cytokine-responsive transcription factor binding site (121). Invertebrates have cytokine-like proteins similar to the interleukins and tumor necrosis factors of vertebrates (122). Transcription factor binding sites in the 5′-flanking sequence demonstrate a bipartite distribution of LPS- and inflammatory cytokine-responsive elements that are strikingly similar to that described for murine iNOS gene promoters (123, 124). Studies of *Drosophila* NOS regulation have shown (125) that insect NOS activity is solely dependent on Ca^2+^ and calmodulin, like the constitutive vertebrate NOSs. Although the activity of *Drosophila* NOS is very low compared to other NOSs, low amount of NO produced may be sufficient for functioning as a signaling molecule (126).

As already commented, NO is rather a signaling than cytotoxic molecule in invertebrates. In vertebrates, as exemplified by rodent macrophages, high amounts of NO are produced as a result of activation of iNOS with LPS and IFNγ. This is a part of defense armor, which otherwise acts as a double edge sword. However, stimulation of macrophages only with LPS results in a low NO production and less than 15% of cells is iNOS positive. IFNγ enhanced LPS-induced secretion of NO by recruiting increasingly greater numbers of macrophages into the production of iNOS (127). The gene of iNOS is synergistically activated by LPS and IFNγ (123, 124). The iNOS promotor contains two important regions termed RI and RII. The effects of LPS stimulation are mediated by elements in both RI and RII, whereas IFNγ functions through RII only (123, 124). In addition, IFNγ alone is not able to activate through RII, acting solely to augment the effect of LPS on RI (123). A variety of LPS response elements, including NF-κB, occurs and the NF-κB site in LPS-mediated transcriptional activation of iNOS is required (128). Perhaps the increase of frequency of LPS responsive cells in effect of IFNγ action, and consequent NO production is the key factor to enhance NO formation. It leads to M1 type of response.

TGF-β1 seems to be the most potent regulator of iNOS. In natural killer cells, neutrophils and macrophages, TGF-β1 diminishes iNOS activity, influencing gene expression, mRNA stability and translation, and NOS protein stability (129). For suppression of LPS-stimulated iNOS in bone marrow-derived mouse macrophages, both Smad2 and Smad3 are required. Down regulation of iNOS mRNA undergoes by suppressing the IRF3- IFNβ-STAT1 pathway (130). Mutual feedback regulation between iNOS and TGF-β1 is also possible, as latent TGF-β1 can be activated by exogenous NO (131).TGF-β appears to be important endogenous mediator that keeps resident/wound healing macrophages in M2 dominant mode. A decrease in TGF-β production in macrophages brought about “activation” of these cells. Similarly, removing of TGF-β from cell culture (coming from serum added), caused much more NO produced, and less synergy between LPS and IFN-γ in stimulation of NO production (1).

## Concluding Remarks

**Figure 1:**
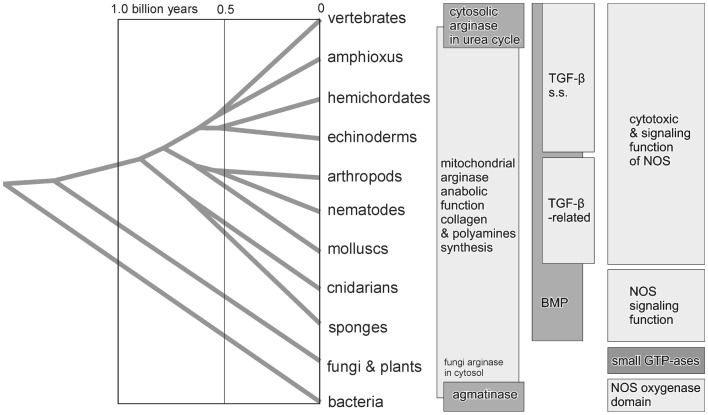
**Proposed order of appearance of arginase, TGF-β superfamily of proteins, and nitric oxide synthase (NOS), superimposed on the metazoan phylogenetic tree, implies a pattern of polarization of vertebrate macrophage to M1 and M2-types of activation**.

Sophisticated ways of signaling observed in contemporary vertebrates show how complex are results of molecular evolution. Although specialization of macrophage responses is based on two ancient mechanisms: cytotoxic activity of iNOS and anabolic function of arginase (Figure 1). It is suggested here that not defense against infection but rather the TGF-β-signaling was at the origin of the M1/M2 macrophage specialized functions. Such signaling is known to operate already in primitive invertebrates, both in their embryonic development and wound healing. A prototypic inducer of M1 response bacterial LPS, alone activates production of only a small amount of NO by iNOS-type enzyme and generates signal propagation through cGMP cyclase in invertebrates. It remains unknown, to what degree invertebrate analogs of IFNγ would be able to enhance LPS-induced NO production, as no experimental data about enhancement of NO production by IFNγ-like cytokine in *Drosophila* are available. Presumably, the ability of M1 macrophages to produce large amounts of NO in response to microbial infection is a vertebrate evolutionary invention, known to be present already at the fish grade (132). At this stage the arginase function in M2 macrophages, inherited after invertebrate ancestors, was to deliver ornithine for processes of extracellular matrix synthesis, of importance in organogenesis and wound healing. The latter serves also as a protection against metazoan parasites. Thus, the main function of M2 macrophages is originally connected with the TGF-β superfamily of proteins, which in primitive metazoans are involved in regulation of tissue and organ differentiation in embryogenesis. Looking back in evolution also indicates that both NOS/NO and arginases/ornithine are primitive innate responses in macrophages that long preceded the development of T and B cells (adaptive immunity).

## Conflict of Interest Statement

The author declares that the research was conducted in the absence of any commercial or financial relationships that could be construed as a potential conflict of interest.
